# Monocytosis at the time of diagnosis has a negative prognostic impact in myelodysplastic syndromes with less than 5% bone marrow blasts

**DOI:** 10.1007/s00277-022-05043-y

**Published:** 2022-11-21

**Authors:** A. Kasprzak, C. Assadi, K. Nachtkamp, M. Rudelius, R. Haas, A. Giagounidis, K. Götze, N. Gattermann, U. Germing

**Affiliations:** 1 Department of Hematology, Oncology and Clinical Immunology, Heinrich-Heine University, Moorenstr. 5, 40225 Düsseldorf, Germany; 2 Department of Anesthesiology, Marienhospital, Aachen, Germany; 3 Department of Pathology, Ludwig Maximilian University, Munich, Germany; 4 Department of Hematology, Oncology and Palliative Care, Marienhospital, Düsseldorf, Germany; 5 Department of Hematology and Oncology, Klinikum Rechts Der Isaar, Munich, Germany

**Keywords:** Myelodysplastic syndromes, Monocytosis, Prognosis

## Abstract

The prognostic impact of monocytosis has not yet been determined in patients with myelodysplastic syndromes (MDS). We examined absolute monocyte counts in the peripheral blood at the time of diagnosis in 1949 patients with a bone marrow blast count < 5%, a condition we call MDS < EB1 (MDS with a blast percentage lower than that of MDS with excess blasts 1, according to the WHO classification). Monocytosis (> 600/µl) was associated with higher median hemoglobin, WBC, and ANC, and more favorable karyotype (*p* = .001). Nevertheless, monocytosis was associated with shorter overall survival (OS) (108 vs. 126 months, *p* = .002) and earlier transformation into AML (*p* < .001). In patients with sideroblastic phenotype, the percentage of ring sideroblasts significantly correlated with the monocyte count (*p* = .005), and OS was significantly shorter when monocytosis was documented (88 vs. 132 months, *p* = .004). The survival disadvantage of patients with MDS < EB1 and peripheral blood monocytosis suggests that these patients suffer from a CMML-like disease. Even though they are generally classified as MDS with persistent monocytosis, such patients should be considered candidates for therapeutic options employed in CMML.

## Introduction

Myelodysplastic syndromes (MDS) are a group of clonal stem cell disorders characterized by dysplasia of bone marrow cells and peripheral cytopenia. MDS have a highly heterogeneous presentation: while patients with low-risk disease according to the revised international prognostic scoring system (IPSS-R) [1] have a rather indolent course of disease, patients with high-risk disease may rapidly exhibit symptoms of bone marrow insufficiency and may ultimately experience progression to acute myeloid leukemia (AML).

Monocytes are an essential part of the host defence system. They are primed for phagocytosis, innate immune responses, migration to inflammation sites, and cytokine secretion. This functional diversity might be explained by the fact that the monocyte population is not homogenous, but can be divided into smaller, more distinct subsets. Proliferation of monocytes occurs in reactive conditions during infectious diseases, as well as in myeloid neoplasms with clonal monocytosis, such as chronic myelomonocytic leukemia (CMML).

Before 2001, CMML was classified as MDS due to its MDS-like features like dysplasia of bone marrow (BM) cells. However, in contrast to other MDS subtypes, a diagnosis of CMML requires relative and absolute peripheral monocytosis. According to the WHO 2016 classification of myeloid neoplasms [2], CMML is considered a distinct entity with a unique clinical presentation. Classical CMML can be diagnosed if the following diagnostic criteria are met: absolute monocytosis in the peripheral blood (pb) (> 1 × 10/L), provided that monocytes represent > 10% of pb leukocytes, absence of a reactive condition or myeloproliferative neoplasm that may explain persistent monocytosis, a bone marrow blast count < 20%, and exclusion of Philadelphia chromosome, PMF, ET, PV, or mutations in the PDGFRA or PDGFRB, FGFR1, and PCM1-JAK2 genes [3]. Combined with dysplastic features of at least one lineage of myeloid blood cells, the minimal diagnostic criteria for CMML are met.

Recently, it became evident that myeloid neoplasms may evolve from precursor stages that are characterized by clonal hematopoiesis but do not produce noticeable symptoms. Accordingly, conditions like clonal hematopoiesis of indeterminate potential (CHIP) or clonal cytopenia of undetermined significance (CCUS) lack significant dysplasia, have neither cytogenetic aberrations nor elevated blast counts, and do not meet the diagnostic criteria for classical myeloid neoplasms [4]. In the case of absolute and relative monocytosis without dysplasia, the condition is called idiopathic monocytosis of unknown significance (IMUS) [5]. Another entity in this group of precursor stages is oligomonocytic myelomonocytic leukemia (O-CMML) [6]. Here, monocytosis does not reach the threshold required for the diagnosis of classical CMML, while the other diagnostic prerequisites are met. Most of these patients show typical features of CMML, like dysplasia in bone marrow cells, organomegaly, and molecular aberrations known to be CMML-related (e.g., alterations in ASXL1, TET2, and SRSF2) [7]. Some O-CMMLs may later transform into overt CMML or even into secondary AML (sAML).

In daily practice, differentiation between classical MDS and classical CMML may be challenging. We often encounter patients who present with persistent monocytosis but do not meet the criteria for CMML or any of the aforementioned conditions. These patients have usually been classified as MDS with monocytosis or as a form of myeloproliferative disease. Since the distinction between CMML and MDS is clinically relevant and has potential impact on therapeutic options, better characterization of these cases is needed. Hence, 1949 MDS patients in our cohort were retrospectively analyzed to determine the impact that peripheral monocytosis might have on the prognosis of patients with no excess blasts in the bone marrow (“MDS < EB1”).

## Methods

For this retrospective analysis, we retrieved from our Düsseldorf MDS registry the follow-up data of 1949 patients with classical MDS and less than 5% bone marrow blasts. Patients were diagnosed between 1988 and 2019. Patients with more than 5% bone marrow blasts, high-risk patients according to the IPSS-R, and patients with MDS del(5q) as well as patients fulfilling WHO diagnostic criteria for any CMML-subtype were excluded. MDS was diagnosed and classified according to the WHO 2016 classification of myeloid neoplasms. Patients were then assigned to two groups: those with peripheral monocytosis at the time of diagnosis (group A), and those with normal monocyte count (group B). According to the normal range for peripheral blood monocyte counts in the central laboratory of the University Hospital Düsseldorf, monocytosis was defined as a monocyte count > 600/µl but less than 1000/µl, in order to rule out undiagnosed CMML. Patients with monocyte counts between 200 and 600/µl were considered to have physiological values. Within the scope of our analysis, we examined patients with monocytopenia (< 200 monocytes/µl), as well.

Frequencies for categorial parameters were displayed using cross-tabulation, and differences were calculated using Student’s *t*-test. Differences in continuous variables were assessed with the Mann–Whitney *U* test.

OS for the entire cohort was calculated as the time from the first diagnosis to death from any cause or last follow-up date. Time-to-event curves were calculated using the Kaplan–Meier method along with log-rank tests for univariate analyses. Multivariate analyses were performed using the Cox regression model.

In all analyses, a *p*-value < 0.05 was considered to be statistically significant. Statistical analyses were performed using IBM SPSS (SPSS Inc. Chicago, IL) and GraphPad Prism 9 (GraphPad Software Inc.).

## Results

### Baseline features

Our study cohort of 1949 patients with < 5% blasts in the bone marrow included 856 females (43.9%) and 1093 males (56.1%). The median age at initial diagnosis was 70 years (range 18–99 years) in the entire cohort, and the median duration of follow-up was 23 months (1–500 months). Based on the 2016 WHO classification, distribution among MDS types was as follows: 255 (13.1%) MDS-SLD, 1362 (69.9%) MDS-MLD, 242 (12.4%) MDS-RS-SLD, and 90 (4.6%) MDS-RS-MLD. The distribution did not differ significantly between patients with normal monocyte count at the time of diagnosis and patients with peripheral monocytosis (p = 0.895, Table 1).

**Table 1:** Comparison of baseline characteristics between patients with (group A) and without (group B) peripheral blood monocytosis

	Monocytosis Group A		Without monocytosis Group B		*p*-value
	*n*	%	*n*	%	
	326	16.7	1623	83.3	
Age, years (median, range)	74 (32–99)		71 (18–100)		.002
Gender					.002
Male	213	65.3	880	54.2	
Female	113	34.7	742	45.7	
MDS subtype					.895
MDS-SLD	43	13.2	212	13.1	.876
MDS-MLD	219	67.2	1143	70.4	.836
MDS-RS-SLD	49	15.0	193	11.9	.757
MDS-RS-MLD	15	4.6	75	4.6	.898
Peripheral blood cell count					
Hb level g/dl (median, range)	10.0 (3.6–15.7)		9.3 (2.2–16.9)		< .001
ANC/µl (median, range)	3993 (0–8079)		1638 (0–5202)		< .001
Platelets/µl (median, range)	120,000 (7000–1,504,000)		119,000 (2000–154,000)		.797
WBC count/µl (median, range)	6800 (1300–85,000)		3700 (100–44,360)		< .001
pb monocyte count/µl (median, range)	1114 (601–8845)		184 (0–600)		< .001
Karyotype according to IPSS-R					.023
Very good	9	2.8	47	2.9	
Good	98	30.0	452	27.8	
Intermediate	19	5.8	147	23.6	
Poor	15	4.6	48	3.0	
Very poor	11	3.4	62	3.8	
Follow-up, months (median, range)	25 (0–199)		30 (0–528)		

The majority of patients (*n* = 1623, 83.3%) had normal peripheral blood monocyte counts (200–600/µl). The median was 222/µl in the whole group. The group included 326 patients with peripheral monocytosis > 600/µl (16.7%).

Monocyte counts differed significantly between patients with and without sideroblastic phenotype, with a median count of 248/µl in patients with ring sideroblasts and 214/µl in patients without RS (*p* < 0.001).

In order to compare comorbidities at the time of diagnosis, we employed the MDS comorbidity index (MDS CI) but could not detect a difference between patients with normal vs. elevated monocyte count. In both groups, cardiac diseases and malignant tumors were the most frequent comorbidities. According to the MDS CI, 56 patients (36.8%) in the monocytosis group had a low-risk comorbidity score, 72 patients (47.4%) belonged to the intermediate-risk group, and 24 patients (15.8%) had a high risk according to the MDS CI. Neither the distribution of comorbidities nor the MDS CI categories differed significantly between groups A and B (*p* = 0.832 and *p* = 0.803).

In the entire study cohort, 504 patients (55.5%) presented with a normal karyotype at baseline, while 404 patients (44.5%) had an abnormal karyotype, according to the cytogenetic risk groups defined by the IPSS-R. In group A with patients with monocytosis, the majority of patients had a normal karyotype (93 patients, 61.6%). In group B without monocytosis, a normal karyotype was also found in most of the cases (411 patients, 54.3%). When comparing the specific IPSS-R subtypes, patients with monocytosis had a more favorable karyotype than patients with a physiologic monocyte count (*p* = 0.023). Detailed patient characteristics are given in Table 1.

#### Peripheral blood counts strongly correlate with peripheral monocytosis

Given the limited data on MDS patients with monocytosis, we aimed to identify differences between group A and group B and thus analyzed peripheral blood counts at the time of diagnosis. We detected significant differences regarding all three hematopoietic lineages (Table 1). Elevated peripheral monocyte counts were associated with higher median hemoglobin, WBC, and ANC. This finding was corroborated by positive correlations between monocyte counts and the above-mentioned parameters.

#### Cytogenetic aberrations strongly correlate with peripheral monocytosis

Patients with peripheral monocytosis presented with a more favorable karyotype according to the IPSS-R, compared to patients with normal monocyte count (Table 1). Most patients’ cytogenetics had a good prognosis (*n* = 98, 30%). Intermediate, poor, and very poor risk cytogenetics according to the IPSS-R were less frequent with 5.8% (*n* = 19), 4.6% (*n* = 15), and 3.4% (*n* = 11). Karyotypes with very good prognosis were the least frequent with 2.8% (*n* = 9). Monocytosis was more frequent in MDS patients with a favorable karyotype (*p* = 0.001).

### Prognostic parameters

#### Impact of increased peripheral monocytes on median overall survival

**Fig. 1:**
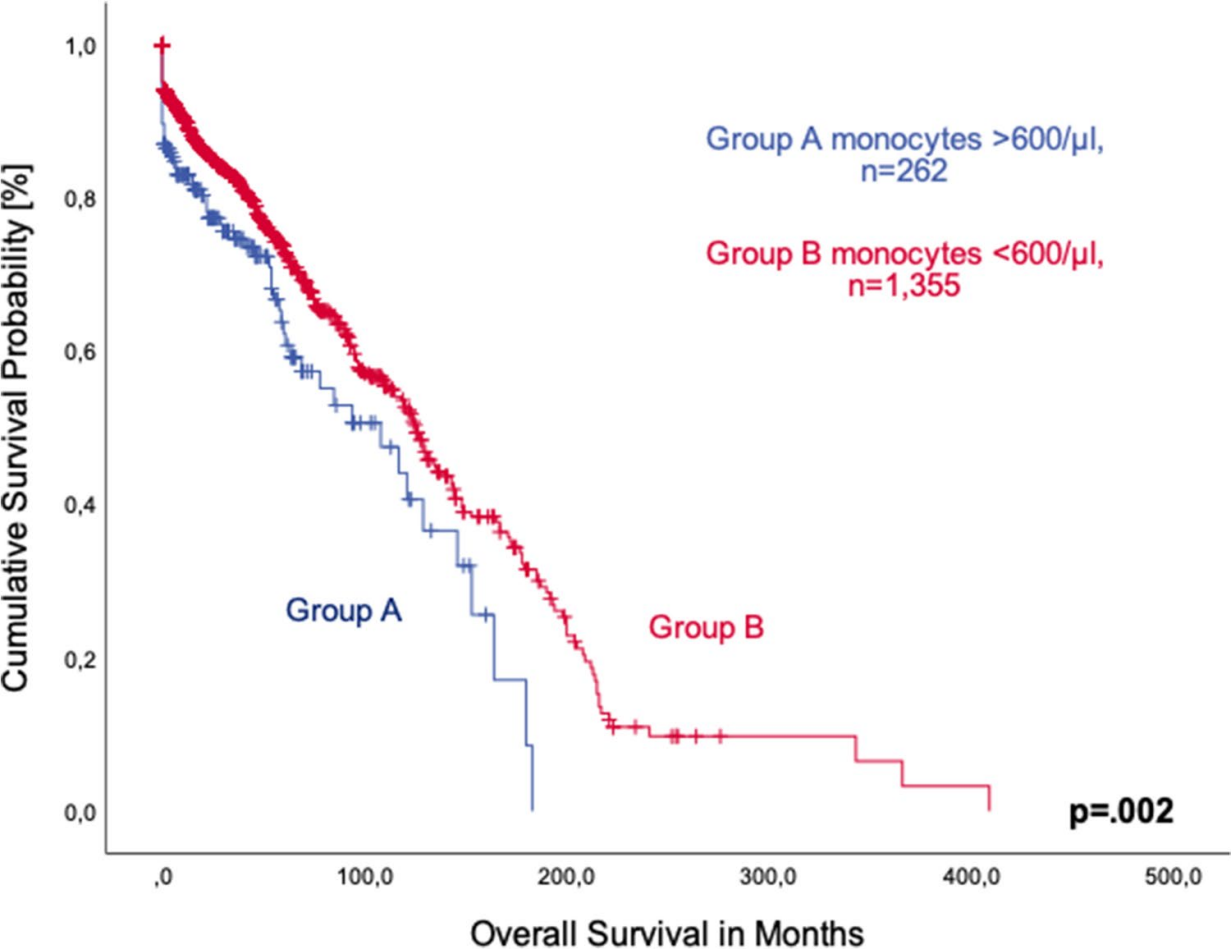
Comparison of overall survival of patients with MDS-SLD/MDS-MLD with (group **A**) and without peripheral monocytosis (group **B**)

A large proportion of patients with MDS-SLD and MDS-MLD presented with a monocyte count below 600/µl (*n* = 1355, 69.5%). Only 262 patients (13.4%) showed monocytosis at the time of diagnosis. We found that patients with monocytosis had significantly shorter median overall survival (108 months, 95%CI 64.6–151.4 vs. 126 months, 95%CI 113.9–138.1; *p* = 0.002, Fig. 1).

**Fig. 2:**
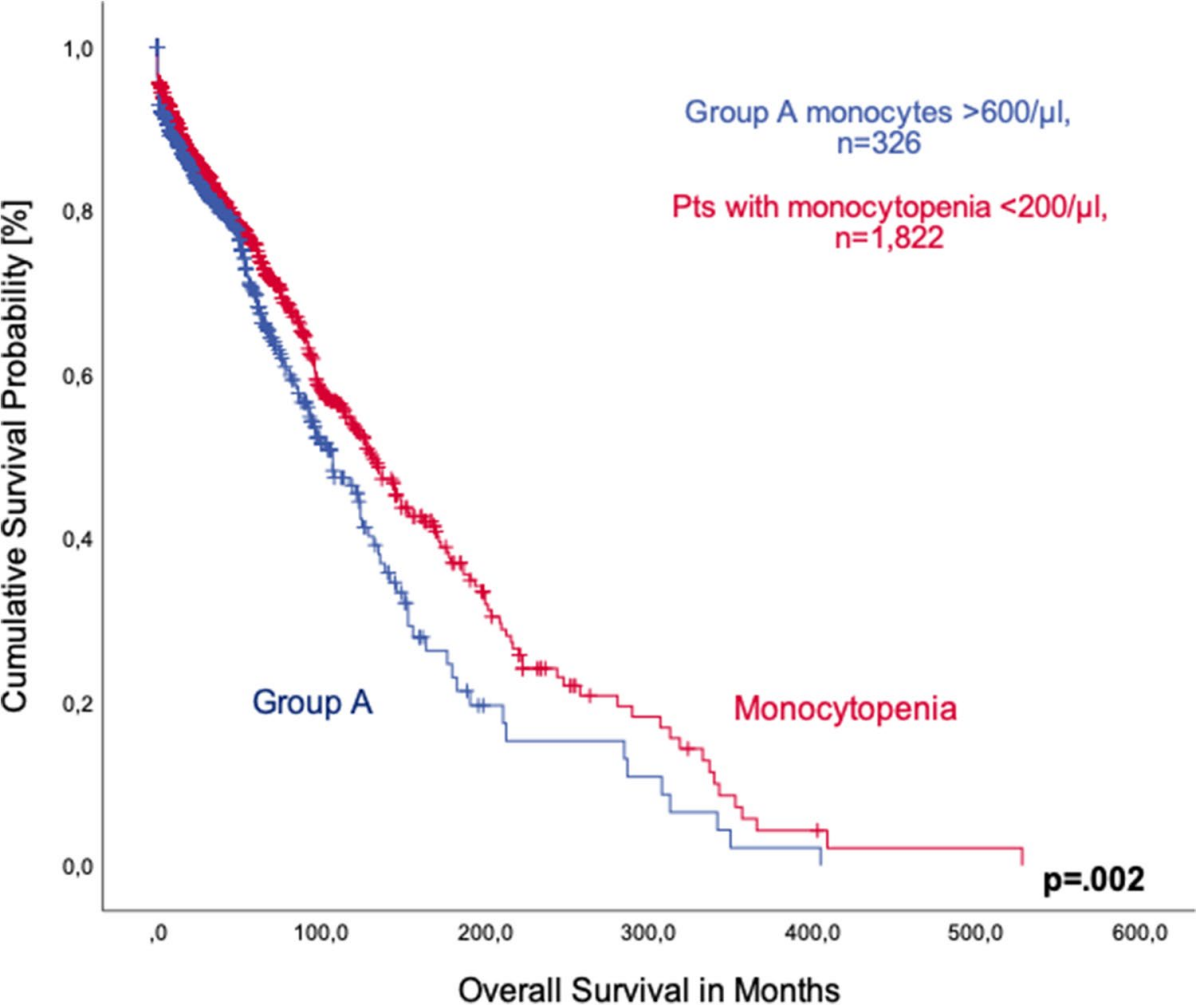
Comparison of overall survival of MDS patients with peripheral monocytosis (group **A**) and monocytopenia

We also compared 1822 patients with monocytopenia (< 200/µl) from our registry and patients showing monocytosis. Again, patients with monocytosis had shorter median overall survival (107 months, 95%CI 89.4–124.5 vs. 132 months, 95%CI 116.0–147.9; *p* = 0.002, Fig. 2).

We examined a group of 332 patients with sideroblastic phenotype, of whom 64 (19.3%) presented with peripheral monocytosis at the time of diagnosis, while 268 patients (80.7%) showed normal monocyte counts. A significant positive correlation was detected between monocyte count and diagnosis of MDS with ring sideroblasts (*p* = 0.005).

**Fig. 3:**
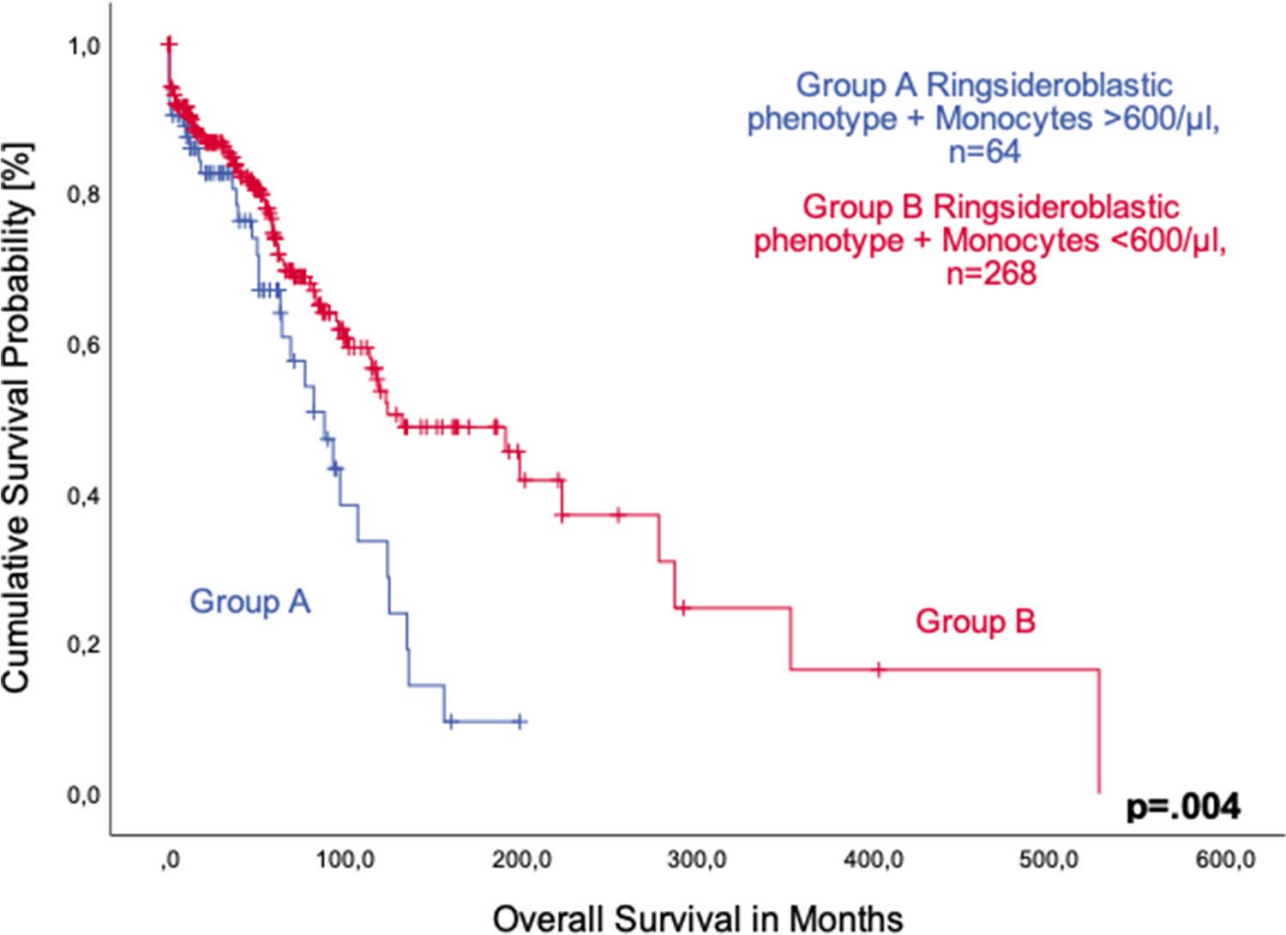
Comparison of overall survival in patients with sideroblastic phenotype with (group **A**) and without monocytosis (group **B**)

**Fig. 4:**
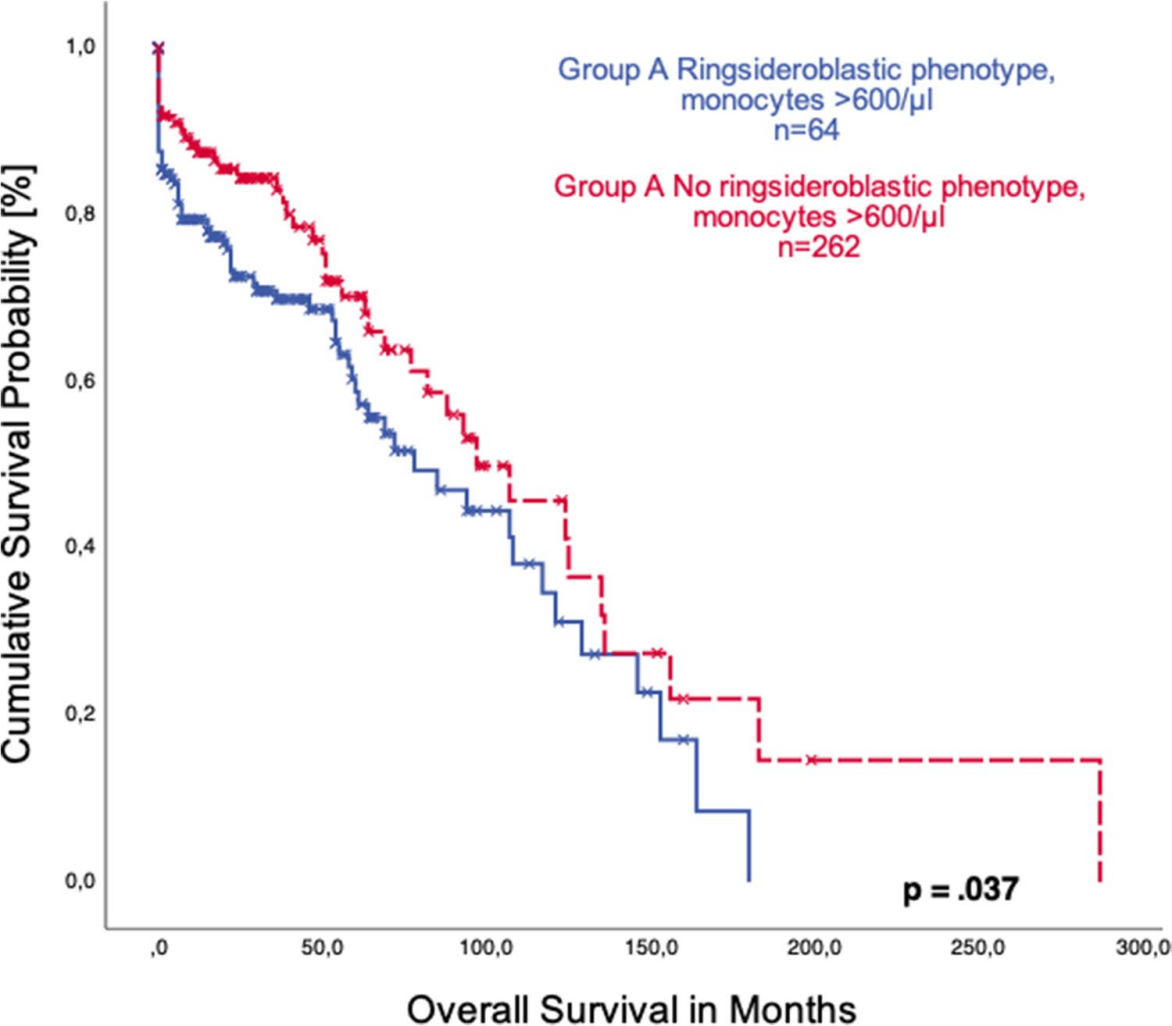
Comparison of overall survival in monocytotic patients (group **A**) with and without sideroblastic phenotype

Of note, patients with normal monocyte count had a significantly longer survival than those with monocytosis, with a median overall survival of 132 months (95%CI 72.4–191.6) vs. 88 months (95%CI 61.0–114.9), respectively (*p* = 0.004, Fig. 3). Patients with a combination of monocytosis and ring sideroblasts had a shorter survival than monocytotic patients without ring sideroblasts (median OS 78 months, 95%CI 48.4–107.6 vs. 97 months, 95%CI 62.7–131.3; *p* = 0.037, Fig. 4).

We finally compared our monocytotic patients with two control groups from our MDS Registry. The first control group comprised 697 patients with classical CMML according to WHO 2016 criteria. Interestingly, patients in our study group had a shorter median OS than patients with CMML. Patients with monocytosis lived for a median of 87 months (95%CI 79.0–99.9). CMML patients had a median overall survival of 91 months (95%CI 86.7–116.2). However, the difference was statistically not significant (*p* = 0.749).

The second control group was based on patients with high-risk disease, i.e., 905 patients with MDS EB1 and 1580 patients with MDS EB2. As expected, patients with high-risk disease had inferior overall survival (68 months (95%CI 91.3–110.8) vs 87 months (95%CI 79.0–99.9; *p* = 0.186).

### Mutation analysis

#### Association between monocyte count and mutations in the epigenetic regulator TET2

We found somatic mutations in 64 patients with monocytosis (group A). Of these, 39 were related to epigenetic regulation (TET2, IDH 1/2, DNMT3A, ASXL1), 22 involved splicing factors (SF3B1, SRSF2, U2AF1), and three affected the transcription factor RUNX1. Interestingly, TET2 was the only mutated gene that showed a strong correlation with monocytosis (*p* < 0.001).

Monocytotic patients with a TET2 mutation (*n* = 18) had a shorter survival than those without a TET2 mutation (*n* = 5) with 85 vs. 185 months, respectively. Due to the small number of patients, the difference did not reach statistical significance (*p* = 0.061).

Mutational analysis of spliceosome-related genes was available in 163 patients. Patients with ring sideroblasts had significantly more spliceosome mutations (*p* < 0.001). In the entire group of patients with ring sideroblasts, 114 (69.9%) had a spliceosome mutation. The majority showed a somatic mutation in SF3B1 (*n* = 107, 93.8%). Nine patients (7.9%) had a mutant SRSF2 gene, and five (4.4%) had a mutation in U2AF1. The distribution among spliceosome mutations was significantly different between patients with and without ring sideroblasts.

#### Impact of increased monocyte count on cumulative risk and time to transformation into AML

Thirty-two patients (9.8%) in group A suffered transformation into AML. In group B, a comparable proportion (*n* = 153, 9.5%) showed evolution to AML. As our analysis did not yet reach the median, a statistically significant difference was not detectable using the log-rank test (*p* = 0.115). However, the Wilcoxon test implied that patients with peripheral monocytosis (> 600/µl) showed earlier transformation into AML (*p* < 0.001).

## Discussion

Based on the Düsseldorf MDS Registry, we report on 1949 MDS patients with < 5% bone marrow blasts who did not fulfil the diagnostic criteria of CMML. In these patients with MDS < EB1, pb monocytosis at the time of diagnosis was associated with less severe cytopenias, more favorable karyotypes, and more frequent somatic mutations in the TET2 gene. Our results also suggest that increased peripheral blood monocytes are an adverse prognostic factor for OS and transformation into sAML. To the best of our knowledge, this study is the first to examine the impact of monocytosis in MDS.

It has previously been shown that MDS may evolve into classical CMML, a development that portends a poor prognosis. Schuler et al. [8] found that MDS patients with significant bone marrow monocytosis, but without increased peripheral monocyte count, are more likely to develop CMML according to WHO criteria than patients without bone marrow monocytosis. Patients exhibiting a CMML-like phenotype in the bone marrow also had a higher incidence of constitutional symptoms. Another study on bone marrow monocytosis [9] confirmed these findings and concluded that an increase in mature monocytes in the bone marrow is associated with inferior prognosis in patients with all types of MDS. Since our study included only patients without excess blasts, patient characteristics differed substantially between our cohort and the two aforementioned studies. Nevertheless, there are important similarities.

We observed significantly higher ANC and hemoglobin in monocytotic MDS, indicating that the respective patients have less severe cytopenias. This is in accordance with generally higher blood counts in patients with CMML compared to classical MDS.

Interestingly, we identified a positive correlation between pb monocytosis and a more favorable karyotype. This is in line with the finding that the majority of patients with classical CMML have a normal karyotype [10].

Somatic mutations of TET2 are frequent and usually occur as early clonal events in patients with myeloid neoplasms, especially CMML. In a large population-based study of clonal hematopoiesis, somatic mutations in TET2 and other epigenetic regulators were found in 11% of the population older than 80 years. These mutations were associated with an increased risk of hematological malignancies and greater mortality [11].

Up to now, mutations in TET2 have not been proven to independently impact OS or leukemia-free survival in patients with CMML [12]. In our cohort, patients with TET2 mutations had inferior OS. However, due to small patient numbers, this result did not reach statistical significance.

Mutations in genes coding for spliceosome components, especially SF3B1, are characteristic for patients with ring sideroblasts. The 2016 WHO classification of myeloid neoplasms has included SF3B1 mutations among the diagnostic criteria for MDS types with ring sideroblasts. Alterations in spliceosome genes are known to mediate a myeloproliferative environment [13]. Interestingly, we observed a correlation between the sideroblastic phenotype and peripheral blood monocytosis. Our finding that MDS patients with ring sideroblasts and monocytosis have a shorter survival than patients without monocytosis corroborates the generally unfavorable prognostic impact of spliceosome mutations.

Remarkably, patients with MDS < EB1 and monocytosis showed less severe cytopenias and a more favorable karyotype at the time of initial diagnosis. Hence, these patients are less prone to suffer from disease-related symptoms like fatigue or bleeding. Yet, we showed that they have an inferior prognosis compared to classical MDS patients because evolution to secondary AML occurred earlier. This finding may be due to the expansion of an existing MDS subclone in patients with monocytosis. Such a clone with predominantly monocytic differentiation may proliferate vigorously and may thus be particularly prone to acquire additional mutations, faster than usual in patients without excess blasts. As early clonal events, TET2 mutations are well-positioned to drive this development.

## Conclusion

The survival disadvantage of MDS patients with peripheral monocytosis at the time of diagnosis, regardless of the presence or absence of ring sideroblasts, suggests that these patients have a CMML-like disease. The prognosis is worse, despite higher blood counts and more favorable karyotypes. We propose that such patients should be screened for other features typically found in CMML and should thus undergo bone marrow analysis including cytomorphology, esterase staining, and mutation analysis. Even though they are classified as MDS with persistent monocytosis, such patients should be considered candidates for therapeutic options employed in patients with CMML.
